# Ambient air pollution and thyroid function in Spanish adults. A nationwide population-based study (Di@bet.es study)

**DOI:** 10.1186/s12940-022-00889-1

**Published:** 2022-08-17

**Authors:** Sergio Valdés, Viyey Doulatram-Gamgaram, Cristina Maldonado-Araque, Ana Lago-Sampedro, Eva García-Escobar, Sara García-Serrano, Marta García-Vivanco, Luis Garrido Juan, Mark Richard Theobald, Victoria Gil, Fernando Martín-Llorente, Pilar Ocon, Alfonso Calle-Pascual, Luis Castaño, Elías Delgado, Edelmiro Menendez, Josep Franch-Nadal, Sonia Gaztambide, Joan Girbés, F Javier Chaves, José L Galán-García, Gabriel Aguilera-Venegas, Carolina Gutierrez-Repiso, José Carlos Fernández-García, Natalia Colomo, Federico Soriguer, Eduardo García-Fuentes, Gemma Rojo-Martínez

**Affiliations:** 1 Department of Endocrinology and Nutrition, Hospital Regional Universitario de Málaga/Universidad de Málaga, Instituto de Investigación Biomedica de Málaga-IBIMA, Málaga, Spain; 2 Centro de Investigación Biomédica en Red de Diabetes y Enfermedades Metabólicas Asociadas (CIBERDEM), Instituto de Salud Carlos III, Madrid, Spain; 3 Centro de Investigaciones Energéticas, Medioambientales y Tecnológicas (CIEMAT) - División de Contaminación Atmosférica, Madrid, Spain; 4 UGC de Laboratorio (Bioquímica), Hospital Regional Universitario de Málaga, Instituto de Investigación Biomédica de Málaga (IBIMA), Málaga, Spain; 5 Department of Endocrinology and Nutrition and Instituto de Investigación, Department Medicine II, Sanitaria University Hospital S. Carlos (IdISSC), Universidad Complutense (UCM), Madrid, Spain; 6 Hospital Universitario Cruces, UPV/EHU, BioCrucesBarakaldo, Spain; 7 Centro de Investigación Biomédica en Red de Enfermedades Raras (CIBERER), Instituto de Salud Carlos III, Madrid, Spain; 8 Department of Endocrinology and Nutrition, Hospital Universitario Central de Asturias / University of Oviedo, Instituto de Investigación Sanitaria del Principado de Asturias (ISPA), Oviedo, Spain; 9 EAP Raval Sud, Institut Català de la Salut, Unitat de Suport a la Recerca (IDIAP – Fundació Jordi Gol), Red GEDAPS, Primary Care, Barcelona, Spain; 10 Genomic Studies and Genetic Diagnosis Unit, Fundación de Investigación del Hospital Clínico de Valencia - INCLIVA, Valencia, Spain; 11 Department of Applied Mathematics, University of Málaga, Málaga, Spain; 12 UGC de Endocrinología y Nutrición. Hospital Universitario Virgen de la Victoria, Instituto de Investigación Biomedica de Málaga-IBIMA, Málaga, Spain; 13 CIBER de Fisiopatología de la Obesidad y Nutrición (CIBEROBN), Instituto de Salud Carlos III, Madrid, Spain; 14 Málaga Academy of Sciences, Málaga, Spain; 15 Unidad de Gestión Clínica de Aparato Digestivo, Hospital Universitario Virgen de la Victoria, Instituto de Investigación Biomédica de Málaga - IBIMA, Málaga, Spain; 16 CIBER Enfermedades Hepáticas y Digestivas - CIBEREHD, Instituto de Salud Carlos III, Málaga, Spain

**Keywords:** Air pollution, Thyroid, Spain

## Abstract

**Background:**

Recent reports have suggested that air pollution may impact thyroid function, although the evidence is still scarce and inconclusive. In this study we evaluated the association of exposure to air pollutants to thyroid function parameters in a nationwide sample representative of the adult population of Spain.

**Methods:**

The Di@bet.es study is a national, cross-sectional, population-based survey which was conducted in 2008-2010 using a random cluster sampling of the Spanish population. The present analyses included 3859 individuals, without a previous thyroid disease diagnosis, and with negative thyroid peroxidase antibodies (TPO Abs) and thyroid-stimulating hormone (TSH) levels of 0.1-20 mIU/L. Participants were assigned air pollution concentrations for particulate matter <2.5μm (PM_2.5_) and Nitrogen Dioxide (NO_2_), corresponding to the health examination year, obtained by means of modeling combined with measurements taken at air quality stations (CHIMERE chemistry-transport model). TSH, free thyroxine (FT4), free triiodothyronine (FT3) and TPO Abs concentrations were analyzed using an electrochemiluminescence immunoassay (Modular Analytics E170 Roche).

**Results:**

In multivariate linear regression models, there was a highly significant negative correlation between PM_2.5_ concentrations and both FT4 (p<0.001), and FT3 levels (*p*<0.001). In multivariate logistic regression, there was a significant association between PM_2.5_ concentrations and the odds of presenting high TSH [OR 1.24 (1.01-1.52) *p*=0.043], lower FT4 [OR 1.25 (1.02-1.54) *p*=0.032] and low FT3 levels [1.48 (1.19-1.84) *p*=<0.001] per each IQR increase in PM_2.5_ (4.86 μg/m^3^). There was no association between NO_2_ concentrations and thyroid hormone levels. No significant heterogeneity was seen in the results between groups of men, pre-menopausal and post-menopausal women.

**Conclusions:**

Exposures to PM_2.5_ in the general population were associated with mild alterations in thyroid function.

## Background

Air pollution is recognized as the largest environmental health risk worldwide [1–3]. A large proportion of the world population lives in places where the air quality standards for the protection of human health are exceeded. Spain is not an exception to this global problem. In fact during 2019, 98% and 66% of the monitoring stations in Spain recorded levels of PM_2.5_ and NO_2_ which were above the current WHO standard guidelines [4]

Air pollution exposure has been linked to a wide range of adverse health outcomes including cardiovascular disease, chronic obstructive pulmonary disease, lung cancer, type 2 diabetes, impaired cognitive function and adverse pregnancy outcomes [1–3]. Oxidative stress, immune and inflammatory responses and epigenetic changes are some of the underlying factors mediating these adverse effects [5].

Increasing the knowledge about the potential emerging health effects of air pollution is essential for a better assessment of the global burden of disease attributable to these hazards [1].

Accordingly, recent reports, focused mainly on pregnancy and birth cohorts [6–12], but also on the general adult population [13–15], have suggested that air pollution may have an impact on thyroid function, although the evidence in this regard is still scarce and inconclusive. This association may however be of potential global health importance, since air pollution exposure is widespread, and thyroid hormones play essential roles in growth and development, and affect virtually every organ system, including the heart, the nervous system, the bones, the gastrointestinal system and metabolism.

Therefore, in the present study we aimed to investigate whether the exposure to air pollutants [particles with an aerodynamic diameter of less than 2.5 microns (PM_2.5_), and Nitrogen Dioxide (NO_2_)] was associated with thyroid function parameters in a nationwide sample representative of the adult population of Spain.

## Methods

### Study design, setting and population

The Di@bet.es Study is a national, cross-sectional, population-based study which was conducted in 2008-2010 using a random cluster sampling of the Spanish population [16]. The study sample consisted of 5072 not pregnant adults (> 18 years), randomly selected from the National Health System registries distributed into 110 clusters (primary health care centers). Thyroid function studies [thyroid-stimulating hormone [TSH], free thyroxine (FT4), free triiodothyronine (FT3) and thyroid peroxidase antibodies (TPO Abs)], were performed in 90% of this sample [17]. Individuals with missing data did not differ in age, sex or other characteristics of interest from individuals with complete data.

For the present analysis, we excluded all the subjects with a previous thyroid disease diagnosis, and/or taking interfering medications (levothyroxine, thionamides, amiodarone or lithium). We also excluded individuals with a positive TPO Abs test (≥ 50 IU/ml), or with very high (>20 mIU/L) or suppressed (<0.1 mIU/L) TSH levels.

This research was carried out in accordance with the Declaration of Helsinki of the World Medical Association [18]. Written informed consent was obtained from all the participants. The study was approved by the Ethics and Clinical Investigation Committee of the Hospital Regional Universitario de Málaga (Málaga, Spain) in addition to other regional ethics and clinical investigation committees all over Spain.

### Variables and procedures

The participants were invited to attend a single examination visit at their health center. Information was collected by means of an interviewer administered structured questionnaire, followed by a physical examination and blood sampling. Information on age, gender, educational level (none/basic/high school/college), and smoking habit (current, former or never been smokers) was obtained by questionnaire. Medical history and medications were also recorded. Menopause was considered in women who reported more than 12 months of amenorrhea without any other obvious pathological or physiological cause. Weight and height were measured and the body mass index (BMI) was calculated by standardized methods.

Blood samples were obtained in fasting conditions, were immediately centrifuged and the serum was frozen until analysis. A casual urinary sample was also collected and samples were frozen until analysis. Samples were managed by the biochemistry laboratory of the Hospital Regional Universitario de Málaga, the IBIMA Biobank and by the CIBERDEM Biorepository (IDIBAPS Biobank). TSH, FT4, FT3 and TPO Abs concentrations were analyzed using an electrochemiluminescence immunoassay (Modular Analytics E170, Roche Diagnostics, Basel, Switzerland). The functional sensitivity of the TSH assay was 0.014 mIU/L. The intra-assay coefficients of variation were: TSH, 1.5–1.2%; FT4 1.8–1.6%; FT3 1.3–2.0% and TPOAb 4.8–2.8%. The inter-assay coefficients of variation for the low and high levels of serum TSH, FT4, FT3 and TPO Abs quality control materials were 3.5 and 2.7%, 4.17 and 2.64%, 3.78 and 2.21%, and 8.5 and 5.2%, respectively. All samples were analyzed at the biochemistry laboratory of the Hospital Regional Universitario de Málaga. Urinary iodine (UI) was analyzed using the modified method of Benotti and Benotti [19]. The intra and inter-assay coefficients of variation of UI assay were 2.01% and 4.53%, respectively. The UI assay was subjected to a program of external quality assessment for the determination of iodine in urine of the Spanish Association of Neonatal Screening (AECNE) and of Ensuring the Quality of Iodine Procedures (EQUIP) Program. All UI samples were analyzed in the Research Laboratory of the Hospital Regional Universitario de Málaga (Spain).

### Exposure assessment

Modeled mean annual PM_2.5_ and NO_2_ concentrations in Spain for the period 2008 to 2010 were calculated with the CHIMERE chemistry-transport model [20]. This model calculates the concentration of gaseous species and both inorganic and organic aerosols of primary and secondary origin, including primary particulate matter, mineral dust, sulphate, nitrate, ammonium, secondary organic species and water. This model has been broadly evaluated in Spain by comparison with measured air pollutants at a large set of monitoring sites [21, 22]. The model was applied to a domain covering the Iberian Peninsula at a horizontal resolution of 0.1x0.1º. The modelled concentrations were corrected with observed values, by considering a methodology described by Martín et al [23] in which 1) a bias is calculated with regard to the observations in the Spanish air quality network of monitoring sites, 2) these biases are spatially interpolated using a krigging methodology to obtain a gridded bias, and 3) this gridded bias is applied to the modelled concentration grid. This methodology considers a different bias grid for rural and urban sites that are then combined and weighted by population density. We assigned the average annual exposure to air pollutants corresponding to the health examination year of each participant by interpolating the estimated concentrations to the centroid of their residential postal codes.

Data on mean annual temperature (°C) from each municipality of residence were obtained from the Spanish National Meteorological Agency [24]

### Statistical analysis

We applied linear regression models to assess associations between air pollutant and thyroid hormone levels (TSH, FT4, FT3), which were log transformed to normalize distributions and also to limit the influence of extreme values. First, we explored any indications of non-linearity in the associations using Curve Estimation procedures. None of the models tested improved the linear model and there was no indication of a threshold effect. Association estimates were presented as percent changes with corresponding 95% confidence intervals (calculated by 100 × [exp(b)-1]), per each interquartile range (IQR) increase in air pollutant concentrations which equated to 4.86 μg/m^3^ PM_2.5_, and 12.62 μg/m^3^ NO_2_.

We also used logistic regression models to investigate the associations of ambient air pollutants with high TSH levels (defined as a percentile (p) >95), low FT4 (≤p5) and low FT3 (≤p5). These results are presented as odds ratios (ORs) with corresponding 95% CIs again per each interquartile range (IQR) increase in air pollutants.

All these models were controlled for possible confounders such us age, sex, UI, BMI, education level, smoking status and ambient temperature. In addition, we performed subgroup analyses to test potential effect modifications in subgroups according to sex and menopausal status. Homogeneity of the ORs between subgroups was tested with the Breslow-Day test. All the statistical analyses were performed with IBM SPSS statistics 23.0. Reported p values were based on two-sided tests with statistical significance set at 0.05.

## Results

A total of 3859 individuals were included in the analysis (Table 1). The sample was composed of 1757 men (45.5%), 1100 premenopausal women (28.5%) and 1002 post-menopausal women (26.0%). Mean age of the population was 50.1±17.1 years (range 18–93 years). Mean and median UI concentrations were 132.2±81.6 and 114.9 μg/L respectively, concordant with an iodine sufficient population. Mean and median concentrations of TSH, FT4 and FT3 were within the expected range, taking into consideration the exclusion criteria for this analysis.

**Table 1:** Clinical characteristics of the study sample (3859 individuals free of thyroid disease)

	**%**	**Mean±SD**	**Median**	**Range**
**Age (years)**		50.1±17.1		18-93
**Men**	45.5			
**Premenopausal women**	28.5			
**Postmenopausal women**	26.0			
**Smoking**				
** Current**	26.7			
** Former**	24.1			
** Never**	49.2			
**Education level**				
** No studies**	12.8			
** Basic**	47.5			
** High School-College**	39.7			
**BMI (kg/m**^**2**^**)**		28.0±5.1		12.2-61.3
**UI** (**μg/L)**		132.2±81.6	114.9	1.5-632.5
**TSH (µUI/mL)**		2.33±1.35	2.06	0.11-18.50
**FT4 (pmol/L)**		15.14±2.07	15.05	8.21-26.24
**FT3 (pmol/L)**		5.00±0.73	4.95	2.59-11.95

*BMI* Body mass index. *UI* Urinary iodine.

Table 2 summarizes residential estimates of outdoor air pollution concentrations assigned to the study participants in the year of examination. The median (25^th^-75^th^ percentile) PM_2.5_ and NO_2_ exposure levels were 12.19 (10.50-15.36) and 16.77 (12.28-24.90) μg/m^3^, respectively. Most values were within the current European Ambient Air Quality Directive target values (Directive 2008/50/EC) [25].

**Table 2:** Descriptive statistics for Air Pollutants concentrations (μg/m^3^) in the study sample

	**Percentile**							
**Pollutant**	**5**^**th**^	**25**^**th**^	**50**^**th**^	**75**^**th**^	**95**^**th**^	**Mean**	**Minimum**	**Maximum**
**PM**_**2.5**_	8.14	10.50	12.19	15.36	20.99	12.81	3.44	22.34
**NO**_**2**_	6.44	12.28	16.77	24.90	50.25	20.43	3.58	51.38

*PM*_*2.5*_ Particles with an aerodynamic diameter of less than 2.5 microns, *NO*_*2*_ Nitrogen dioxide.

Table 3 shows the results of the linear correlations between air pollutant concentrations and thyroid hormone levels (TSH, FT4, FT3) in crude, and multivariate adjusted linear regression models. The results showed a small, but highly significant negative correlation between PM_2.5_ concentrations and both FT4 and FT3 levels, which remained after multivariate adjustment of the data. In the fully adjusted model, there was a 0.5 and 0.9% reduction in FT4 and FT3 levels per each IQR increase in PM_2.5_ (4.86 μg/m^3^) . Models were run again after exclusion of potential outliers in the log-scale without any significant changes in the results. There were no associations between NO_2_ and thyroid hormone levels.

**Table 3:** Association between Air pollutants concentrations (μg/m^3^) and thyroid hormones levels (TSH, FT4, FT3)

	**TSH**	**p**	**FT4**	**p**	**FT3**	**p**
	**% change (95% CI)**		**% change (95% CI)**		**% change (95% CI)**	
**PM**_**2.5**_						
Crude	0.2 (-0.8. 1.2)	0.767	**-0.5 (-0.7 .-0.3)**	<0.001	**-0.7 (-1.0. -0.5)**	<0.001
Multivariate	0.2 (-0.8. 1.3)	0.664	**-0.5 (-0.8 .-0.8)**	<0.001	**-0.9 (-1.2. -0.7)**	<0.001
**NO**_**2**_						
Crude	-0.3 (-1.1. 0.4)	0.403	-0.2 (-0.4. 0.0)	0.067	-0.1 (-0.3. 0.1)	0.234
Multivariate	-0.5 (-1.3. 0.4)	0.278	-0.2 (-0.4. 0.0)	0.118	-0.1 (-0.3. 0.1)	0.367

% changes and *p* values calculated by linear regression per interquartile range (IQR) increase in air pollutants concentrations (PM_2.5_: 4.86 μg/m^3^, NO_2_: 12.62 μg/m^3^)

In bold: ß coefficients with *p* values <0.05

^#^Multivariate model: Adjusted to age, sex, urinary iodine, BMI, education level, smoking status and ambient temperature

*PM*_*2.5*_ Particles with an aerodynamic diameter of less than 2.5 microns, *NO*_*2*_ Nitrogen dioxide

Table 4 shows the crude and multivariate-adjusted ORs for presenting high TSH levels (>P95), low FT4 (≤p5) and low FT3 (≤p5) per each IQR increase in air pollutant concentrations. There was a significant association between PM_2.5_ concentrations and the odds of presenting lower FT4 [OR 1.30 (1.08-1.57) p=0.006] and FT3 levels [OR 1.34 (1.11-1.62) p=0.002] per each IQR increase in PM_2.5_ (4.86 μg/m^3^). Again, the association remained after multivariate adjustment. There was also a reciprocal association between PM_2.5_ concentrations and the odds of presenting high TSH levels which reached statistical significance after multivariate adjustment [OR 1.24 (1.01-1.52) p=0.043]. As in the linear model, there was no association between NO_2_ concentrations and thyroid hormone levels in this model.

**Table 4:** Odd Ratios (OR) for presenting high TSH, low FT4 and low FT3 according to Air pollutants concentrations

	**TSH>4.66 µUI/mL (p95)**			**FT4≤11.97 pmol/L (p5)**			**FT3≤3.93 pmol/L (p5)**		
	**OR**	**CI 95%**	**p**	**OR**	**CI 95%**	**p**	**OR**	**CI 95%**	**p**
**PM**_**2.5**_									
Crude	1.20	0.99-1.45	0.060	**1.30**	**1.08-1.57**	0.006	**1.34**	**1.11-1.62**	0.002
Multivariate	**1.24**	**1.01-1.52**	0.043	**1.25**	**1.02-1.54**	0.032	**1.48**	**1.19-1.84**	<0.001
**NO**_**2**_									
Crude	1.13	0.98-1.31	0.094	1.12	0.97-1.29	0.122	1.02	0.88-1.19	0.797
Multivariate	1.13	0.97-1.32	0.115	1.15	0.99-1.35	0.074	1.00	0.84-1.18	0.997

ORs, 95% CI and *p* values were calculated by logistic regression per interquartile range (IQR) increase in air pollutants concentrations (PM_2.5_: 4.86 μg/m^3^, NO_2_: 12.62 μg/m^3^).

In bold: ORs with *p* values <0.05

^#^Multivariate model: Adjusted to age, sex, urinary iodine, BMI, education level, smoking status and ambient temperature

*PM*_*2.5*_ Particles with an aerodynamic diameter of less than 2.5 microns, *NO*_*2*_ nitrogen dioxide

**Fig. 1:**
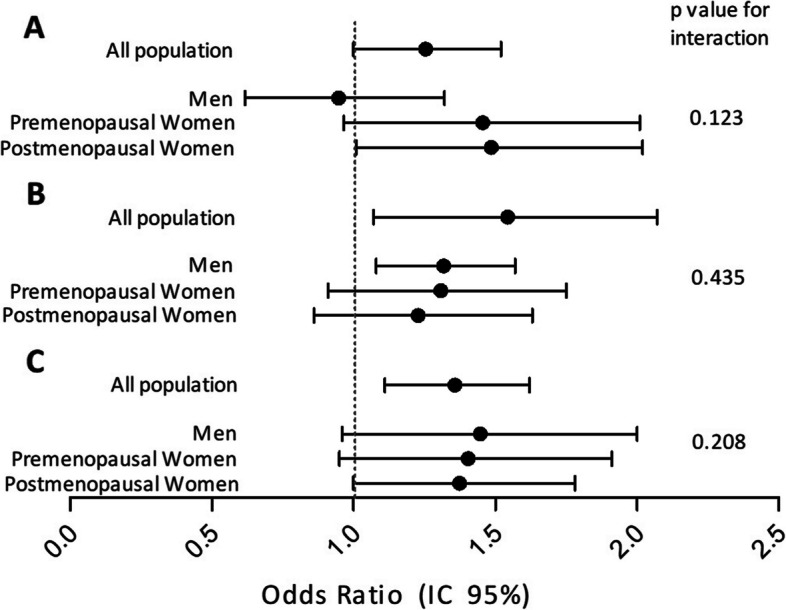
Results of the logistic regression analyses between PM_2.5_ exposures and high TSH (p>95) (**A**), low FT4 (≤p5) (**B**) and low FT3 (≤p5) (**C**) in different population subgroups

Figure 1 shows the results of the logistic regression analyses between PM_2.5_ exposures and high TSH levels (>P95), low FT4 (≤p5) and low FT3 (≤p5) in different population subgroups. Although there were some small differences between groups, no significant heterogeneity was seen in the results between the studied groups (men, pre-menopausal and post-menopausal women).

## Discussion

In this nationwide sample representative of the adult population of Spain we found significant negative associations between exposures to PM_2.5_ and thyroid hormone levels whereas exposures to NO_2_ were not associated with any changes in thyroid function.

These findings are in line with previous studies suggesting that air pollution might impact thyroid function in pregnant women and neonates [6–12], and also in the general adult population [13–15]. In fact, our data almost completely coincide with those of the studies in early pregnancy by Zhao et al [6] and Ghassabian et al [8], showing significant associations between PM_2.5_ exposure and higher odds of maternal hypothyroxinemia. Other studies in general population samples have also shown a negative association between PM_2.5_ and both FT4 and FT3 levels [15] whereas in the recent study from Kim et al [14] in a national sample of Korean adults, there was an association between NO_2_ and CO and reduced FT4 concentration, but this association was not observed with PM_10_. Also, Zaccarelli-Marino et al [13] reported that the odds for hypothyroidism tended to rise when NO_2_, CO, and VOC concentrations increased, whereas this pattern was not observed for PM_10_ and SO_2_. It is of note that both Kim et al [14], and Zaccarelli-Marino et al [13] did not include exposures to PM_2.5_ in their estimations. Although PM mass is well acknowledged as an imperfect metric, PM_2.5_ particles differ to coarse particles in their composition, and are known to have a higher penetration capacity and reactivity. In fact, most evidence supports PM_2.5_ as the principal air pollutant with the greatest threat to global public health [2].

Interestingly, as suggested in other studies [8], our findings also indicate that the associations of air pollution exposure with thyroid function may occur within relatively low PM_2.5_ concentration ranges. In fact, the association we describe in our study occurs within exposure concentration ranges which are well below the existing European Ambient Air Quality Directive target values (PM_2.5_<25 µg/m^3^) [25]. In contrast, the WHO guidelines have recently lowered the PM_2.5_ recommended maximum annual exposure to 5 µg/m^3^ [3].

The underlying mechanisms for this association remain unclear.

Thyroid endocrine disruption could be a possibility. The thyroid hormone system is a main target of endocrine disruptor compounds at all levels [26]. Although, to the best of our knowledge, there is no conclusive evidence linking air pollutants with thyroid disruption, experimental data have demonstrated that these compounds may in fact interfere with other nuclear receptors such as oestrogen receptor signalling [27, 28]. Another potential mechanism could be related to oxidative stress (OS), and inflammatory response, secondary to the inhalation of air pollutants. In fact, OS and activation of pro-inflammatory signaling are hallmarks of the pathophysiological mechanisms underlying air pollution–mediated systemic effects [5, 29]. The fact that the thyroid gland can be damaged by OS has been demonstrated in iodine excess studies [30]. Interestingly, however, the observed differences could also represent an adaptive response. Accordingly, it is well known that thyroid hormones regulate OS and increase reactive oxygen species (ROS) generation by promoting oxidative phosphorylation [31]. IL6, among other cytokines, induces OS and has been shown to induce suppression of the hypothalamic-pituitary axis, and suppresses T3 generation by D1 and D2 deiodinases, in the setting of nonthyroidal illness [30]. In line with this, lower thyroid hormone levels could be interpreted as a compensatory mechanism to buffer ROS damage reactive to air pollutants.

Finally, due to the observational nature of the associations and the lack of toxicological studies we cannot currently explain the full spectrum of the mechanisms underlying these associations and further studies are warranted.

Our study has several strengths, including the population-based design and the inclusion of a complete thyroid function evaluation in all subjects (TSH, FT4, FT3, TPOAbs), as well as other extensive individual-level data of clinical, demographic and lifestyle variables, and iodine status, which allowed us to perform an adequate sample selection, and multivariate adjustment of the data. Also, we have analysed and expanded data to subgroups like men or post-menopausal women whereas most previous data were based on pregnant women or in the general population as a whole. Finally, our nationwide perspective, allows us to extrapolate our results more widely than local or regional studies increasing the public health implications of the findings.

The limitations of our study include its observational cross-sectional nature; therefore, as already mentioned, we cannot establish causal associations or exclude residual confounding in the relation between air pollutants and thyroid function. Also, we used ambient outdoor measurements modelled at the residential addresses of the participants as a proxy for exposure to air pollution, whereas no other relevant information such as time–activity patterns, proximity to main roads and occupational exposures was available. This is however a common limitation to most studies assessing the health effects of air pollution and, in fact, air quality guidelines focus primarily on ambient (outdoor) air pollution for their recommendations [3]. Finally, our exposure models were developed based on yearly exposures to air pollutants, whereas more refined measures to look at different lags were not available.

## Conclusions

In summary, our study reports an association between the exposure to PM_2.5_ and markers of thyroid dysfunction in the general population which is concordant with previous studies. The nature of this association remains unknown. Additional studies are warranted to expand the data in this field. In the meantime, the association of air pollution exposure with thyroid function is of global health importance because air pollution exposure is widespread and its effect on the thyroid could have potential clinical effects on the health of the population.

## Data Availability

The datasets used and/or analysed during the current study are available from the corresponding author on reasonable request.

## Abbreviations

BMI: Body mass index
FT4: Free thyroxine
FT3: Free triiodothyronine
IQR: Interquartile range
NO_2_: Nitrogen Dioxide
OS: Oxidative Stress
P: Percentile
PM_2.5_: Particles with an aerodynamic diameter of less than 2.5 microns
ROS: Reactive Oxygen Species
TPOAbs: Thyroid Peroxidase Antibodies
TSH: Thyroid-stimulating hormone
UI: Urinary iodine
WHO: World Health Organization
